# The three-factor structure of the Autism-Spectrum Quotient Japanese version in pregnant women

**DOI:** 10.3389/fpsyt.2023.1275043

**Published:** 2023-10-31

**Authors:** Ekachaeryanti Zain, Naoki Fukui, Yuichiro Watanabe, Koyo Hashijiri, Takaharu Motegi, Maki Ogawa, Jun Egawa, Koji Nishijima, Toshiyuki Someya

**Affiliations:** ^1^Department of Psychiatry, Niigata University Graduate School of Medical and Dental Sciences, Niigata, Japan; ^2^Department of Psychiatry, Faculty of Medicine, Mulawarman University, Samarinda, Indonesia; ^3^Department of Psychiatry, Uonuma Kikan Hospital, Niigata, Japan; ^4^Department of Obstetrics and Gynecology, Niigata University Graduate School of Medical and Dental Sciences, Niigata, Japan

**Keywords:** Autism-Spectrum Quotient (AQ), autism spectrum disorder, autism spectrum conditions (ASC), perinatal women, factor structure, factor analysis

## Abstract

**Background:**

There is a rising interest in perinatal mental health studies, and proper psychometric tools to assess autistic traits among this population in Japan are vital.

**Objective:**

This study aimed to clarify the optimal factor structure of the AQ as part of a perinatal mental health research project.

**Methods:**

We used the Japanese version of the AQ (AQ-J) to measure autistic-like traits in pregnant women. Participants were 4,287 Japanese women who were pregnant or who had given birth within the last month. We performed exploratory factor analysis (EFA) using the first sample group (n = 2,154) to obtain factor structures for the final item selections. We performed confirmatory factor analysis (CFA) using the second sample group (n = 2,133) to obtain a model with good fit, then compared the model to all previously proposed models to determine the best-fitting model.

**Results:**

The EFA analysis identified a model consisting of 25 items distributed across three factors. Cronbach’s alpha for the total 25-item AQ-J, 9-item “Social interaction” factor, 11-item “Non-verbal communication” factor, and 5-item “Restricted interest” factor was 0.829, 0.829, 0.755, and 0.576, respectively. McDonald’s omega and its 95% confidence interval were 0.826 (0.821–0.836), 0.835 (0.821–0.837), 0.755 (0.744–0.766), and 0.603 (0.556–0.596), respectively. CFA confirmed that the three-factor structure had an acceptable fit (goodness of fit index: 0.900, comparative fit index: 0.860, root mean square error of approximation: 0.066). These findings indicated that the three-factor model was better than the 13 existing models.

**Conclusion:**

The findings are discussed in relation to the adequacy of the AQ-J for assessing autistic traits in perinatal women. We recommend the use of this 25-item, three-factor AQ-J model for this population owing to its superiority to all previous models.

## 1. Introduction

The Autism-Spectrum Quotient (AQ) (1) is a widely used instrument that identifies adult individuals with normal intelligence who may have autistic-like traits. The AQ has frequently been used as a screening instrument for autism spectrum conditions (ASC) or broader phenotypes in the general population (2), as a tool in autism research to explore other, non-clinical traits and behaviors associated with autism spectrum disorder (ASD) (1, 3), and in clinical practice to differentiate between individuals with and without Asperger syndrome (4). Autism is currently considered a state on the continuum from an ASD diagnosis, to ASC, then to normality, and the extent of autistic traits can be quantitatively measured for scientific research into autism etiopathogeneses and for clinical practice to establish early diagnosis and intervention.

Several studies have reported the experiences of mothers with autism during the perinatal period and parenthood (5, 6). One study showed that mothers with autism were more likely to have antepartum and postpartum depression than controls (5). They also tend to experience difficulties and dissatisfaction communicating with healthcare providers during perinatal care and higher rates of difficulty breastfeeding (5). In addition, mothers with autism are more likely to experience higher rates of pregnancy complications, including preterm birth, cesarean and induced delivery, and pre-eclampsia (7). During motherhood, mothers with autism report more parenting difficulties, including lower parenting competence and satisfaction/self-esteem (8), and are more likely to feel isolated and express the desire for increased parenting support (5). Therefore, the AQ could be useful for screening and identifying autistic traits in pregnant women to ensure the provision of more tailored mental health care and support of mothers and children during the perinatal period.

Although the AQ is a widely used measure, its reliability has been questioned (9). After its first publication, several researchers proposed factor structures and models for the AQ (2, 10–20). However, the findings were inconsistent. The proposed models ranged from two- to six-factor structures and the item loadings varied across studies. When the AQ was first developed, a five-factor structure for the measure was proposed that included the factors of social skills, attention switching, attention to detail, communication, and imagination (1). However, this five-factor structure was theoretical rather than empirical, and several studies demonstrated that it had poor fit (2, 10, 11). Some studies generated factor structures using data-driven approaches and a few used theory-driven approaches. Most studies on the structure of the AQ have used statistical analyses featuring only principal component analysis or exploratory factor analysis (EFA), which cannot determine whether the proposed model is a good fit. In addition, the proposed factor structures depend on the extent to which the AQ is used to evaluate the phenomenology related to the autism spectrum, which may or may not be limited to the autism domain.

Given the importance of autism screening in pregnant women to ensure perinatal health and support healthy motherhood, this study aimed to clarify the optimal factor structure of the AQ as part of a perinatal mental health research project. To the best of our knowledge, no studies have examined the factor structure of the AQ for assessing pregnant women. Using a data-driven approach, we aimed to generate factor structures that specifically identified the autistic traits included in the ASD criteria according to the Diagnostic and Statistical Manual of Mental Disorders (DSM), so that this can be used in future research and clinical practice. We included a large sample of perinatal women and used EFA to obtain factor structures of the final item selections and confirmatory factor analysis (CFA) to obtain a model with good fit. In addition, we compared our factor structure with the AQ factor structures found in 13 previous studies to identify the best-fitting model.

## 2. Materials and methods

### 2.1. Participants

This study was part of the Perinatal Mental Health Research Project conducted between March 2017 and March 2021 (21–28). Participants of the present study were 4,287 pregnant Japanese women aged 18 years or older from 34 associated obstetric institutions in Niigata prefecture, Japan. We distributed a large-scale questionnaire to obtain AQ data at the time of project enrollment. We included participants who had returned and completely filled in the AQ questionnaire as part of the mental health project. The AQ data were the same as those used in our previous study (28). We excluded participants with serious physical complications, serious pregnancy complications, and ongoing treatment for psychiatric disorders (e.g., ASD, schizophrenia, depression, bipolar disorder, anxiety disorder, or personality disorder). The present study focused on autistic traits rather than ASD. Although it is possible that women with undiagnosed ASD were inadvertently included in this study, the estimated prevalence of ASD is approximately 1%, and the male-to-female ratio is 3:1 (29). Thus, we assumed that the obstetric sample in the present study was comparable to the general population of women, but not to the general population of men and women.

### 2.2. Measures

The AQ (1) is a self-administered instrument that assesses autistic traits in adults with normal intelligence. It comprises a 50-item questionnaire. Each item comprises a short statement. The AQ consists of five subscales of 10 statements each: social skills, attention switching, attention to detail, communication, and imagination. Items are rated by participants on a four-point scale: 1 (“definitely agree”), 2 (“slightly agree”), 3 (“slightly disagree”), and 4 (“definitely disagree”). In the original AQ scoring, Baron-Cohen et al. (1) used a 0/1 binary scale in which, for some items, responses of 1 and 2 are scored as 1; for other items, responses of 3 and 4 are scored as 1. The total possible score range is 0–50. However, in this study, we used the four-point scale as we anticipated that this would provide more information and would yield more valid EFA results. The Japanese version of the AQ (AQ-J) has been validated in a previous study (4).

We collected data on obstetric factors, including gestational age (trimester when the participants responded to the AQ), parity (primipara or multipara), type of conception (natural conception or others), and pregnancy (single or multiple).

### 2.3. Statistical analyses

We randomly divided participants with AQ-J data into two groups. Using the first group (n = 2,154), we performed an EFA with Promax rotation, obtaining the number of factors from a parallel analysis. The parallel analysis indicated that eight factors or fewer were appropriate. Therefore, EFAs were performed, in which the number of factors was specified as eight or less, respectively. The maximum likelihood method and Promax rotation were used for each EFA. Item retention/deletion decisions were made using the following criteria: (a) items with a factor loading >0.40 were retained; (b) items were not retained if they had dual-factor loadings (defined as loadings >0.40 on two or more factors or differences between the loadings on the first two primary factors of <0.20). As a result, factors 4, 5, 6, 7, and 8 were not retained because fewer than three items loaded on them. We reported both Cronbach’s alpha (α) and McDonald’s omega (ω) and its 95% confidence interval (CI) for the whole scale and each subscale to examine internal consistency reliability.

We used the second group (*n* = 2,133) to perform a CFA using the optimal factor structure as extracted from the EFA. CFAs were performed with two-, three-, and four-factor structures, respectively. The three-factor structure showed the best fit. We used the goodness of fit index (GFI), the comparative fit index (CFI), and the root mean square error of approximation (RMSEA) to identify an acceptable fit (GFI ≥0.90, CFI ≥0.90, and RMSEA ≤0.08) (30) between the models and the data. All statistical analyses were performed using SPSS versions 25 and 29 (IBM Corp., Armonk, NY, United States), and Amos 25.0.0 (IBM Japan, Tokyo, Japan).

## 3. Results

### 3.1. Descriptive statistics

We included all data from 4,287 pregnant women who completed the AQ-J questionnaire and had no missing values. We also included questionnaire data from women who had given birth within the last month. Table 1 shows the descriptive statistics of the participants.

**Table 1:** Characteristics of participants (*n* = 4,287).

Variable	Value	Missing value
**Age** (years)	31.90 ± 4.80	
**Gestational age** (T1/T2/T3)	2,782 / 1,170 / 329	6
**Parity** (primipara/multipara)	2,079 / 2,208	
**Conception** (natural/others)	3,748 / 433	106
**Pregnancy** (single/multiple)	4,166 / 47	74
**AQ 25-item scores**		
Total	16.70 ± 6.90	
Social interaction subscale	3.69 ± 2.74	
Non-verbal communication subscale	3.48 ± 2.64	
Restricted interest subscale	0.61 ± 0.99	

Several data are expressed as mean ± standard deviation.

AQ, Autism-Spectrum Quotient; T1, 12–15 weeks of pregnancy; T2, 30–34 weeks of pregnancy; T3, 4 weeks after childbirth.

### 3.2. Exploratory factor analyses

We performed EFA using data from the first group (n = 2,154). Although the parallel analysis indicated that eight factors or fewer were appropriate, the EFA and Promax rotation results indicated that three factors should be retained according to the item retention/deletion criteria. Table 2 shows the EFA results for the AQ-J data. Using the criteria described in the Methods section, we excluded 21 items (1, 2, 4, 7, 15, 18, 21, 24, 25, 26, 28, 29, 30, 33, 34, 35, 40, 41, 43, 49, 50) with factor loadings <0.4, excluded 4 items (5, 12, 20, 23) with dual-factor loadings, and retained 25 items (3, 6, 8, 9, 10, 11, 13, 14, 16, 17, 19, 22, 27, 31, 32, 36, 37, 38, 39, 42, 44, 45, 46, 47, 48) with factor loadings >0.4 without dual-factor loadings. For the 25-item AQ-J, α = 0.829 and ω = 0.826 (95% CI = 0.821–0.836). Factor 1 (“Social interaction”) comprised nine items (10, 13, 17, 22, 38, 44, 46, 47, 48), with α = 0.829 and ω = 0.835 (95% CI = 0.821–0.837). Factor 2 (“Non-verbal communication”) comprised eleven items (3, 8, 11, 14, 27, 31, 32, 36, 37, 42, 45), with α = 0.755 and ω = 0.755 (95% CI = 0.744–0.766). Factor 3 (“Restricted interest”) comprised five items (6, 9, 16, 19, 39), with α = 0.576 and ω = 0.603 (95% CI = 0.556–0.596). Table 3 shows the three-factor structure and the items.

**Table 2:** Exploratory factor analyses of the AQ data (*n* = 2,154).

Item No.	Statement	Factor coefficient		
		Factor 1	Factor 2	Factor 3
1	I prefer to do things with others rather than on my own.	0.378	−0.139	0.004
2	I prefer to do things the same way over and over again.	−0.107	−0.056	0.174
**3**	**If I try to imagine something, I find it very easy to create a picture in my mind.**	0.066	**0.448**	0.059
4	I frequently get so strongly absorbed in one thing that I lose sight of other things.	0.070	−0.077	0.309
5	I often notice small sounds when others do not.	−0.165	0.321	**0.452**
**6**	**I usually notice car number plates or similar strings of information.**	−0.010	0.163	**0.652**
7	Other people frequently tell me that what I’ve said is impolite, even though I think it is polite.	0.055	−0.280	0.297
**8**	**When I’m reading a story, I can easily imagine what the characters might look like.**	−0.059	**0.465**	0.050
**9**	**I am fascinated by dates.**	0.058	0.073	**0.645**
**10**	**In a social group, I can easily keep track of several different people’s conversations.**	**0.554**	0.260	0.007
**11**	**I find social situations easy.**	0.088	**0.513**	−0.001
12	I tend to notice details that others do not.	−0.054	**0.530**	0.366
**13**	**I would rather go to a library than a party.**	**−0.535**	0.184	−0.043
**14**	**I find making up stories easy.**	−0.137	**0.530**	0.181
15	I find myself drawn more strongly to people than to things.	0.394	0.070	−0.015
**16**	**I tend to have very strong interests, which I get upset about if I cannot pursue.**	0.010	−0.097	**0.445**
**17**	**I enjoy social chit-chat.**	**0.622**	0.085	−0.052
18	When I talk, it is not always easy for others to get a word in edgeways.	0.189	−0.139	0.365
**19**	**I am fascinated by numbers.**	0.027	0.110	**0.691**
20	When I’m reading a story, I find it difficult to work out the characters’ intentions.	0.125	**−0.449**	0.282
21	I do not particularly enjoy reading fiction.	0.110	−0.171	0.161
**22**	**I find it hard to make new friends.**	**−0.659**	−0.051	0.014
23	I notice patterns in things all the time.	−0.121	0.356	**0.422**
24	I would rather go to the theater than a museum.	0.169	−0.055	−0.033
25	It does not upset me if my daily routine is disturbed.	0.082	0.161	−0.316
26	I frequently find that I do not know how to keep a conversation going.	−0.312	−0.288	0.228
**27**	**I find it easy to “read between the lines” someone is talking to me.**	−0.045	**0.614**	−0.053
28	I usually concentrate more on the whole picture, rather than the small details.	0.171	−0.027	−0.072
29	I am not very good at remembering phone numbers.	−0.039	−0.281	−0.181
30	I do not usually notice small changes in a situation, or a person’s appearance.	0.014	−0.336	−0.106
**31**	**I know how to tell if someone listening to me is getting bored.**	0.216	**0.460**	0.009
**32**	**I find it easy to do more than one thing at once.**	0.049	**0.494**	−0.024
33	When I talk on the phone, I’m not sure when it’s my turn to speak.	−0.212	−0.291	0.198
34	I enjoy doing things spontaneously.	0.396	0.164	0.043
35	I am often the last to understand the point of a joke.	−0.080	−0.354	0.239
**36**	**I find it easy to work out what someone is thinking or feeling just by looking at their face.**	−0.023	**0.615**	0.088
**37**	**If there is an interruption, I can switch back to what I was doing very quickly.**	0.018	**0.433**	−0.226
**38**	**I am good at social chit-chat.**	**0.750**	0.110	0.053
**39**	**People often tell me that I keep going on and on about the same thing.**	0.013	−0.209	**0.411**
40	When I was young, I used to enjoy playing games involving pretending with other children.	0.153	0.068	−0.007
41	I like to collect information about categories of things (e.g., types of car, types of bird, types of train, types of plant, etc.).	−0.048	0.139	0.386
**42**	**I find it difficult to imagine what it would like to be someone else.**	0.059	**−0.562**	0.118
43	I like to plan any activities I participate in carefully.	−0.067	0.150	0.203
**44**	**I enjoy social occasions.**	**0.832**	−0.090	0.035
**45**	**I find it difficult to work out people’s intentions.**	−0.084	**−0.578**	0.100
**46**	**New situations make me anxious.**	**−0.436**	−0.069	0.096
**47**	**I enjoy meeting new people.**	**0.739**	−0.094	0.057
**48**	**I am a good diplomat.**	**0.890**	−0.028	0.125
49	I am not very good at remembering people’s date of birth.	−0.067	−0.228	−0.185
50	I find it very easy to play games with children that involve pretending.	0.270	0.173	0.059

Values with factor loadings of ≥ 0.40 are shown in bold, and items with no dual-factor loadings are shown in bold.

AQ, Autism-Spectrum Quotient.

**Table 3:** Factor structure and items on the 25-item Autism-Spectrum Quotient (*n* = 2,154).

Factor	AQ items
**Factor 1**	10. In a social group, I can easily keep track of several different people’s conversations.
Social interaction	13*. I would rather go to a library than a party.
(9 items)	17. I enjoy social chit-chat.
	22*. I find it hard to make new friends.
	38. I am good at social chit-chat.
	44. I enjoy social occasions.
	46*. New situations make me anxious.
	47. I enjoy meeting new people.
	48. I am a good diplomat.
**Factor 2**	3. If I try to imagine something, I find it very easy to create a picture in my mind.
Non-verbal communication	8. When I’m reading a story, I can easily imagine what the characters might look like.
(11 items)	11. I find social situations easy.
	14. I find making up stories easy.
	27. I find it easy to “read between the lines” someone is talking to me.
	31. I know how to tell if someone listening to me is getting bored.
	32. I find it easy to do more than one thing at once.
	36. I find it easy to work out what someone is thinking or feeling just by looking at their face.
	37. If there is an interruption, I can switch back to what I was doing very quickly.
	42*. I find it difficult to imagine what it would like to be someone else.
	45*. I find it difficult to work out people’s intentions.
**Factor 3**	6*. I usually notice car number plates or similar strings of information.
Restricted interest	9*. I am fascinated by dates.
(5 items)	16*. I tend to have very strong interests, which I get upset about if I cannot pursue.
	19*. I am fascinated by numbers.
	39*. People often tell me that I keep going on and on about the same thing.

*Reverse-scored items.

AQ, Autism-Spectrum Quotient.

### 3.3. Confirmatory factor analyses

**Figure 1:**
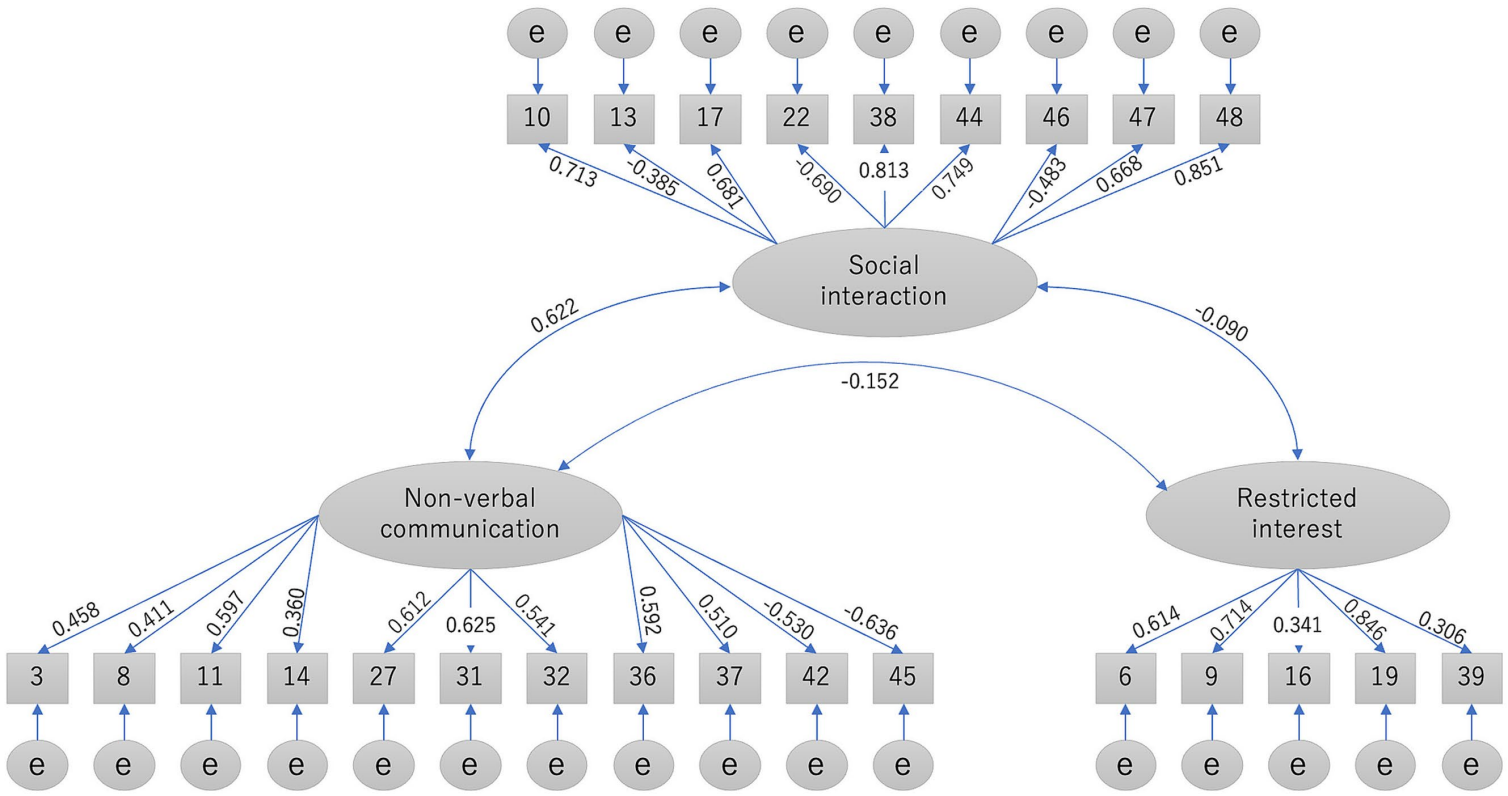
Confirmatory factor analysis of the three-factor model (*n* = 2,154).

We performed CFA using data from the second group (*n* = 2,133). Figure 1 shows the standardized coefficients indicating the association between each item and factor in the CFA. Of the three factors, Restricted interest negatively correlated with Social interaction (*r* = −0.090) and Non-verbal communication (*r* = −0.152). Social interaction positively correlated with Non-verbal communication (*r* = 0.622). The three-factor structure was confirmed to have an acceptable fit for the AQ-J data according to the GFI (0.900) and RMSEA (0.066), but not according to the CFI (0.860).

### 3.4. Comparison with other models

We performed CFA to compare the factor models of previous studies with our second set of AQ-J data (Table 4). A good-fitting model found in a study by Hoekstra et al. (13) could not be compared with the models found in other studies because it was a single study that used the factors as observable variables in the model, whereas other studies used the factors as latent variables. The data from two studies (18, 20) could not be analyzed using CFA because the items overlapped between factors. Therefore, we excluded these three models from our comparison. Table 4 shows that among the remaining 11 factor models, the six-factor structure of Zhu et al. (2) and the present three-factor structure showed the best fit to the data (GFI = 0.913 and 0.900, CFI = 0.802 and 0.860, RMSEA = 0.059 and 0.066, respectively).

**Table 4:** Confirmatory factor analysis of the factor models of previous studies with the second set of AQ data (*n* = 2,133).

No	Study	Year	Country	Original AQ dataset (n), female (%), mean age (years)	No. of factors	Factors	Items	No. of items	No. of total items	GFI	CFI	RMSEA
1	Baron-Cohen et al.	2001	UK	Clinical: AS/HFA (58), 22.4, 31.6	5	Social skill	1, 11, 13, 15, 22, 36, 44, 45, 47, 48	10	50	0.743	0.628	0.067
				Non-clinical: random adults (174), 56.3, 37.0		Attention switching	2, 4, 10, 16, 25, 32, 34, 37, 43, 46	10				
				Non-clinical: unversity students (840), 45.9, 21.0		Attention to detail	5, 6, 9, 12, 19, 23, 28, 29, 30, 49	10				
				Non-clinical: mathematics olympiad winners (16), 6.25, 17.4		Communication	7, 17, 18, 26, 27, 31, 33, 35, 38, 39	10				
						Imagination	3, 8, 14, 20, 21, 24, 40, 41, 42, 50	10				
2	Austin et al.	2005	UK	Non-clinical: undergraduate students (201), 60.6, 20.9	3	Social skills	11, 13, 15, 17, 22, 26, 34, 38, 40, 44, 47, 50	12	26	0.886	0.787	0.068
						Attention to details/patterns	5, 6, 9, 12, 19, 23, 25, 43	8				
						Communication/mindreading	7, 20, 35, 37, 39, 45	6				
3	Hurst et al.	2007	America	Non-clinical: university students (1,005), 77.5, 19.3	5	Social skill scale	1, 11, 13, 15, 22, 36, 44, 45, 47, 48	10	50	0.743	0.628	0.067
						Attention switching scale	2, 4, 10, 16, 25, 32, 34, 37, 43, 46	10				
						Imagination scale	3, 8, 14, 20, 21, 24, 40, 41, 42, 50	10				
						Attention to detail scale	5, 6, 9, 12, 19, 23, 28, 29, 30, 49	10				
						Communication scale	7, 17, 18, 26, 27, 31, 33, 35, 38, 39	10				
4	Hoekstra et al.ª	2008	Netherlands	Non-clinical: university students (961), 60.2, 21.1	2	Social interaction	1, 11, 13, 15, 22, 36, 44, 45, 47, 48 (social skill)	10	50	0.991	0.983	0.064
				Non-clinical: parents (302), 52.9, 35.6			2, 4, 10, 16, 25, 32, 34, 37, 43, 46 (attention switching)	10				
				Clinical: AS/HFA (12), OCD (12), SAD (12), 16.6, ranged 19–57			7, 17, 18, 26, 27, 31, 33, 35, 38, 39 (communication)	10				
							3, 8, 14, 20, 21, 24, 40, 41, 42, 50 (imagination)	10				
						Attention to details	5, 6, 9, 12, 19, 23, 28, 29, 30, 49	10				
5	Stewart and Austin	2009	Scotland	Non-clinical: university students (536), 42.9, 24.3	4	Socialness	1, 11, 13, 15, 17, 18, 22, 26, 38, 44, 46, 47	12	43	0.766	0.650	0.071
						Patterns	5, 6, 9, 12, 19, 23, 29, 41	8				
						Understanding Others/Communication	2, 7, 10, 20, 21, 27, 30, 31, 32, 33, 35, 36, 37, 39, 45, 48	16				
						Imagination	3, 4, 8, 14, 40, 49, 50	7				
6	Kloosterman et al.	2011	America	Non-clinical: university students (522), 85.4, 21.0	5	Social skills	1, 11, 15, 17, 22, 38, 44, 47	8	28	0.880	0.783	0.066
						Communication/mindreading	10, 27, 31, 36, 45	5				
						Restricted/repetitive behavior	2, 4, 18, 25, 39	5				
						Imagination	3, 8, 20, 21, 40	5				
						Attention to detail	5, 6, 12, 19, 23	5				
7	Russell-Smith et al.	2011	Australia	Non-clinical: university students (362), 75.9, 18.7	3	Social skills	1, 10, 11, 13, 15, 17, 22, 26, 34, 38, 44, 46, 47	13	28	0.811	0.762	0.079
				Non-clinical: university students (639), 69.3, 19.1		Details/Patterns	5, 6, 9, 12, 19, 23, 41	7				
						Communication/Mindreading	20, 27, 31, 35, 36, 39, 45, 48	8				
					4	Social Skills	1, 11, 13, 15, 17, 22, 26, 34,38, 44, 46, 47	12	38	0.830	0.712	0.068
						Details/Patterns	5, 6, 9, 12, 16, 19, 23, 29, 41	9				
						Understanding Others/Communication	10, 20, 27, 30, 31, 32, 35, 36, 37, 45	10				
						Imagination	3, 4, 8, 14, 21, 40, 50	7				
8	Hoekstra et al.	2011	Netherlands	Non-clinical: parents and students (1,263), 58.5, 28.4	2	Social behavior	1, 11, 13, 15, 22, 44, 47 (social skills)	7	28	0.878	0.763	0.068
				Non-clinical: general population (1,121), 32.3, 45.6			2, 25, 34, 46 (routine)	4				
				Non-clinical: university students (1,838), 59.9, 20.9			4, 10, 32, 37 (switching)	4				
				Clinical: AS (274), 42.7, 35.3			3, 8, 14, 20, 36, 42, 45, 50 (imagination)	8				
						Numbers and patterns	6, 9, 19, 23, 41	5				
9	Lau et al.	2013	Australia	Non-clinical: general population (314), 77.3, 40.7	5	Sociability	1, 11, 13, 15, 17, 22, 26, 38, 44, 46, 47, 48, 50	13	39	0.846	0.754	0.064
				Clinical: ASD (141), 69.5, 40.5		Social Cognition	8, 10, 20, 27, 28, 31, 32, 36, 37, 42, 45	11				
						Narrow Focus	4, 5, 7, 12, 16, 23, 39	7				
						Interest in Patterns	6, 9, 19, 41	4				
						Resistance to Change	2, 25, 34, 43	4				
10	Freeth et al.	2013	UK	Non-clinical: university students (723), 63.6, 22.3	4	Social situation enjoyment	1, 11, 13, 15, 17, 22, 26, 38, 44, 46, 47	11	35	0.795	0.720	0.073
						Good attention to detail and poor social communication	7, 10, 20, 27, 30, 32, 33, 36, 42, 45, 48	11				
						Imagination	5, 6, 9, 12, 16, 19, 23, 41	8				
						Social awareness and attention to detail	3, 8, 14, 34, 50	5				
			Malaysia	Non-clinical: university students (271), 55.5, 20.9	4	Social situation enjoyment	(10), 11, 13, 15, 17, 22, 26, 38, 44, 46, 48	11	31	N/A	N/A	N/A
						Good attention to detail and poor social communication	6, 9, (10), 27, 29, 30, 36, 37, 49	9				
						Imagination	8, 20, 21, 50, 40	5				
						Social awareness and attention to detail	7, 18, 20, 23, 39, 41	6				
			India	Non-clinical: university students (245), 27.3, 21.0	4	Social situation enjoyment	11, 17, 38, 44, 47	5	24	0.839	0.697	0.090
						Good attention to detail and poor social communication	12, 14, 19,23, 27, 31, 32, 36, 37	9				
						Imagination	18, 20, 22, 26, 33,35, 39	7				
						Social awareness and attention to detail	6, 9, 30	3				
11	Lau et al. (AQ Chinese)	2013	Taiwan	Non-clinical: parents of ASD children (1,208), 50, 41.5	5	Socialness	1, 10, 11, 13, 17, 22, 26, 38, 44, 46, 47, 48	12	35	0.849	0.789	0.064
				Non-clinical: parents of TD children (2,984), 50, 43.0		Mindreading	7, 20, 27, 31, 33, 35, 36, 45	8				
						Patterns	6, 9, 19, 29, 41	5				
						Attention to Details	5, 12, 23, 28	4				
						Attention Switching	4, 16, 32, 34, 37, 39	6				
12	Leth-Steensen et al.	2021	Canada	Non-clinical: university students (633), 76.3, 21.2	5	Communication	7, 18, 20, 21, 27, 30, 31, 33, 35, 36, 39, 45	12	39	N/A	N/A	N/A
						Social skills	1, 11, 13, 15, 17, 22, 26, 38, 44, (46), 47	11				
						Attention to detail	5, 6, 9, 12, 19, 23, 29, 41	8				
						Imagination	3, 8, 14, 40, 50	5				
						Attention switching	2, 25, (46)	3				
13	Zhu et al. (Berenbaum model)	2022	US, Netherland, Australia	Non-clinical: university students (1,006), 65.5, 19.0	6	Social anhedonia	1, 13, 15, 17, 44, 47	6	27	0.913	0.802	0.059
				Non-clinical: general population and students (1,263), 58.5, 28.4		Interest in details/patterns	6, 9, 19, 23, 41	5				
				Non-clinical: university students (1,641), 71.6, 21.4		Social cognition	27, 31, 36, 45	4				
						Social discourse convention	7, 18, 39	3				
						Imagination ability	3, 8, 21, 40, 50	5				
						Desire for predictability/routine	2, 25, 34, 43	4				
14	Our study	2023	Japan	Non-clinical: perinatal women (4,287), 100, 31.9	3	Social interaction	10, 13, 17, 22, 38, 44, 46, 47, 48	9	25	0.900	0.860	0.066
						Non-verbal communication	3, 8, 11, 14, 27, 31, 32, 36, 37, 42, 45	11				
						Restricted interest	6, 9, 16, 19, 39	5				

^ª^Not comparable owing to different factors in the model.

AQ, Autism-Spectrum Quotient; AS, Asperger syndrome, HFA, high-functioning autism; OCD, obsessive-compulsive disorder; SAD, social anxiety disorder; TD, typical development; GFI, goodness of fit index; CFI, comparative fit index; RMSEA, root mean square error of approximation; N/A, not available.

## 4. Discussion

The present study used a data-driven approach to generate a factor structure for the AQ-J. The findings showed that a three-factor structure is optimal and has an acceptable fit according to the statistical analyses. This three-factor structure comprises the factors of Social interaction, Non-verbal communication, and Restricted interest. These three factors are included in the two primary characteristics of ASD in the DSM-5 diagnostic criteria. Our Factor 1 and Factor 2 are included in domain A of the DSM-5 (persistent deficits in social communication and social interaction, including non-verbal communicative behaviors), and Factor 3 is included in DSM-5 domain B (restricted, repetitive patterns of behavior, interests, or activities) (31). These findings are consistent with those of a previous study, which proposed that a three-factor model comprising “Social skill,” “Patterns/details,” and “Communication/mindreading” subscales was the best way to measure specific types of autistic traits using the AQ (9).

The comparison with previously proposed models showed that almost all previous models identified the three factors found in the present study. However, the number of factors and the composition of the items vary across studies. The validity of a measure or a factor structure likely depends on its intended purpose (2). Previous studies have had different goals in generating proposed factor structures, including assessing the relationship of autistic traits to personality (10), assessing the relationship of autistic traits to schizotypy traits (15), and identifying a range of psychological constructs that may be relevant not only to ASC but to a wide variety of clinical phenomena related to schizophrenia spectrum and anxiety disorders (2). The three-factor structure identified in the present study will elucidate the expression of autistic traits by the general population of pregnant Japanese women.

Previous studies suggest that the dimensions of our three-factor structure align with the mapping of several dimensions found in previous studies of Western populations. Our Factor 1 (Social interaction) is mostly related to Sociability (17) and Social skills (1, 10). Our Factor 2 (Non-verbal communication) is mostly related to Social cognition (2, 17) and Communication/mindreading (10–12, 15), representing the difficulties experienced by people with ASD traits with theory of mind, or the ability to understand the beliefs, desires, and intentions of others (32, 33). Finally, our Factor 3 (Restricted interest) is mostly related to Attention to detail (1, 10, 12, 15) and Patterns (2, 14, 16, 17).

Using our AQ-J data, we performed CFA to compare the factor models of 13 previous studies with our own findings. None of the models, including our own, showed a good fit, despite the fact that we had a large sample of perinatal women and analyzed a substantial amount of AQ-J data. However, our three-factor model is comparable or slightly superior to a previous six-factor model (2) and is better than other previous models. Moreover, the Cronbach’s alpha and McDonald’s omega for the 25-item, three-factor AQ-J solution obtained in this study indicated good internal consistency and reliability. Therefore, we suggest the use of this 25-item, three-factor alternative AQ-J to assess autistic traits in perinatal women owing to its superiority to all previous models. However, our model was generated using data from a large sample of perinatal women aged 27–37 years; thus, there may be various discrepancies between our findings and those of previous studies with different samples. A previous study of the general population showed that men had substantially higher AQ scores than women, but that age had no substantial effect on AQ scores (13, 16). Moreover, clinical samples with ASD diagnosis show considerably higher scores than non-clinical samples (1, 13, 16, 17). Therefore, our findings may reflect the effect of sex and the use of a non-clinical sample. Furthermore, a study by Power et al. (34) demonstrated that individuals with ASD showed a greater reduction in fecundity because few ever married or had children compared with individuals in the general population. Specifically, men with ASD had a lower fertility rate than women with ASD. This sex-specific effect may be because ASD morbidity impairs the ability to find suitable sexual partners or inhibits biological fertility to a greater extent in men. In addition, male siblings of individuals with ASD had fewer children, whereas female siblings of individuals with ASD showed no substantial difference from the general population. This pattern may reflect sexually antagonistic genes or undiagnosed symptoms in male siblings of individuals with ASD. Considering these previous findings (34), and the fact that our sample consisted of only perinatal women, the variance of non-autistic traits in our data may be greater than the variance of autistic traits in the normal population, which includes both sexes regardless of marital status.

In the present study, the total scores on the 25-item AQ-J for our participants ranged from 0 to 25. However, we did not determine a cutoff point owing to the unavailability of case data. A previous study indicated that the cutoff point for AQ-J scores is >33 (out of 50 items) for Asperger syndrome or high-functioning autism, but the cutoff point would likely be higher for individuals diagnosed with autistic disorder (4). Therefore, additional studies that include clinical groups are needed to determine a cutoff for this 25-item AQ-J, which could be used for screening autistic traits in a non-clinical population of perinatal women. For our data, the score ranges for Social interaction, Non-verbal communication, and Restricted interest were 0–9 (out of 9), 0–11 (out of 11), and 0–5 (out of 5), respectively. There is a growing recognition that autism may be a heterogeneous condition with various clinical presentations and subtypes (9, 35). Regarding this, our findings also showed some negative interfactor correlations, as found in a previous study (9). We found that the Restricted interest factor negatively correlated with the Social interaction and Non-verbal communication factors. This suggests that the Restricted interest factor may not directly contribute positively to the AQ-J accumulated total score. Individuals may have a high score on one of the factors and a low score on the other two factors. Thus, the AQ score obtained would be classified as moderate or not exceeding the cutoff point even though it actually masks a specific autistic trait. In clinical practice, all symptoms may positively indicate autism. A possible problem in autism research, however, is the confirmation of autism using statistical analyses of AQ scores, as the presence of autistic traits may be apparent in some factor scores but hardly noticeable in scores on other factors, and vice versa. Thus, a global interpretation of the total AQ score requires a detailed interpretation of each accumulated factor score to obtain a general account of the presence of autistic traits. The total 25-item AQ-J solution, the Social interaction factor, and the Non-verbal communication factor showed good internal consistency. However, the Restricted interest factor showed poor internal consistency. Therefore, the use of the total AQ-J score and scores on each factor are recommended in research. Moreover, additional studies are needed to identify possible distinct autism subtypes that may clarify why analysis of AQ scores suggests that some autistic traits appear to cancel out other autistic traits within the same factor model. In clinical practice, all autistic traits are assumed to comprise a combination of symptoms that contribute positively to each other in characterizing autism.

The present study has several limitations that require further discussion. First, a problem with the model comparisons was the existence of differences in the set of retained items; moreover, the scoring method on some of the previous instruments (2) used a Likert scale that was different from the original binary scoring system (1) that we used in this study. Second, we did not conduct structured diagnostic interviews to confirm ASD diagnosis. Additional studies should be performed to validate the 25-item solution of AQ-J as a screening instrument for autistic traits in perinatal women. The present results showed that a three-factor structure comprising 25 items was the optimal model. However, given that this study was conducted on perinatal women, it is necessary to evaluate the suitability of the model in other populations (unmarried women, men in the general population, and ASD patients) and to confirm whether 25 items are more useful for screening than 50 items.

## 5. Conclusion

The present findings suggest that our proposed 25-item, three-factor structure of the AQ-J has an acceptable fit and is superior to all other previous models for use with perinatal women. Therefore, it may be the most suitable model to use for perinatal mental health studies of adult populations. Furthermore, we recommend the use of the 25-item AQ-J total score and the scores on each factor in future research.

## Data availability statement

The datasets presented in this article are not readily available because all relevant data are provided in the paper. We are not able to make the underlying data available to readers, because we do not have permission from the participating institutions to do so. Requests to access the datasets should be directed to TS, psy@med.niigata-u.ac.jp.

## Ethics statement

The studies involving humans were approved by the ethics committee of Niigata University (approval number: 2016–0019) and the ethics committees of the participating obstetric institutions. The studies were conducted in accordance with the local legislation and institutional requirements. The participants provided their written informed consent to participate in this study.

## Author contributions

EZ: Conceptualization, Methodology, Formal analysis, Visualization, Writing – original draft. NF: Conceptualization, Data curation, Formal analysis, Funding acquisition, Investigation, Methodology, Writing – original draft. YW: Conceptualization, Methodology, Writing – original draft. KH: Data curation, Writing – review & editing. TM: Data curation, Funding acquisition, Investigation, Writing – review & editing. MO: Data curation, Funding acquisition, Writing – review & editing. JE: Conceptualization, Methodology, Writing – review & editing. KN: Conceptualization, Methodology, Writing – review & editing. TS: Conceptualization, Methodology, Supervision, Writing – review & editing.
